# IFN-β-inducing, unusual viral RNA species produced by paramyxovirus infection accumulated into distinct cytoplasmic structures in an RNA-type-dependent manner

**DOI:** 10.3389/fmicb.2015.00804

**Published:** 2015-08-04

**Authors:** Asuka Yoshida, Ryoko Kawabata, Tomoyuki Honda, Keizo Tomonaga, Takemasa Sakaguchi, Takashi Irie

**Affiliations:** ^1^Department of Virology, Institute of Biomedical and Health Sciences, Hiroshima University, HiroshimaJapan; ^2^Department of Viral Oncology, Institute for Virus Research, Kyoto University, KyotoJapan

**Keywords:** innate immunity and responses, pathogen-associated molecular patterns (PAMPs), stress granules, Sendai virus (SeV), RNA Viruses

## Abstract

The interferon (IFN) system is one of the most important defensive responses of mammals against viruses, and is rapidly evoked when the pathogen-associated molecular patterns (PAMPs) of viruses are sensed. Non-self, virus-derived RNA species have been identified as the PAMPs of RNA viruses. In the present study, we compared different types of IFN-β-inducing and -non-inducing viruses in the context of Sendai virus infection. We found that some types of unusual viral RNA species were produced by infections with IFN-β-inducing viruses and accumulated into distinct cytoplasmic structures in an RNA-type-dependent manner. One of these structures was similar to the so-called antiviral stress granules (avSGs) formed by an infection with IFN-inducing viruses whose C proteins were knocked-out or mutated. Non-encapsidated, unusual viral RNA harboring the 5′-terminal region of the viral genome as well as RIG-I and typical SG markers accumulated in these granules. Another was a non-SG-like inclusion formed by an infection with the Cantell strain; a copyback-type DI genome, but not an authentic viral genome, specifically accumulated in the inclusion, whereas RIG-I and SG markers did not. The induction of IFN-β was closely associated with the production of these unusual RNAs as well as the formation of the cytoplasmic structures.

## Introduction

Eukaryotic cells are equipped with various defense mechanisms to detect and respond to viral infections rapidly. The interferon (IFN) system is one of the most important natural defenses of mammalian cells in the early phase of viral infection. Host cells sense the invasion of viruses by recognizing their pathogen-associated molecular patterns (PAMPs), including the structural characteristics of viral RNAs that differentiate them from cellular RNAs (Akira et al., 2006). Viral RNAs are detected by non-self RNA sensors such as Toll-like receptors (TLR), and a family of cytosolic RNA helicases termed RIG-I-like receptors (RLRs), including retinoic-acid inducible gene-I (RIG-I), melanoma differentiation-associated gene 5 (MDA5), and laboratory of physiology and genetics gene 2 (LGP2). This is followed by the subsequent induction of IFN-β (Gitlin et al., 2006; Kaisho and Akira, 2006; Saito et al., 2007). Autocrine or paracrine IFNs bind to IFN receptors on the cell surface, leading to the expression of 100s of IFN-stimulated genes (ISGs) through the Jak/STAT signaling pathway, which ultimately exerts various antiviral effects (Akira et al., 2006).

Translational arrest is one of the IFN responses of host cells triggered by viral infections. Various eukaryotic translation initiation factor 2 (eIF2) kinases such as protein kinase R (PKR) are activated in response to IFNs, and the accumulation of phosphorylated eIF2α inhibits the translation of both cellular and viral mRNAs (Anderson and Kedersha, 2002; Kedersha and Anderson, 2002; Holcik and Sonenberg, 2005). Cytoplasmic stress granules (SGs), which are the foci of concentrated 48S translation preinitiation complexes and defined by certain marker RNA binding proteins such as T-cell intracellular antigen-1 (TIA-1), TIA-1-related protein (TIAR), and Ras-Gap-SH3 domain-binding protein (G3BP1), are formed under these conditions (Anderson and Kedersha, 2002; Kedersha and Anderson, 2002). Because they contain stable inert mRNA, SGs are believed to serve as temporary sites at which mRNA-protein complexes are stored to pause active translation or be decayed in adjacent processing bodies (Anderson and Kedersha, 2006; Balagopal and Parker, 2009; Buchan and Parker, 2009).

A number of viruses have been shown to induce the formation of SGs in infected cells, and this may be related to the virus-induced shut-off of cellular protein translation. For example, hepatitis C virus, poliovirus, Semliki Forest virus, and mammalian orthoreovirus promote the shut-off of cellular proteins and the assembly of SGs at the early phase of infection, and this is inhibited as the infection progresses (McInerney et al., 2005; White et al., 2007; Qin et al., 2009, 2011; Ariumi et al., 2011; White and Lloyd, 2011; Garaigorta et al., 2012; Panas et al., 2012; Ruggieri et al., 2012; Fitzgerald and Semler, 2013; Pager et al., 2013; Carroll et al., 2014). Two RNA viruses, respiratory syncytial virus and coronavirus, are also known to utilize SGs as part of the machinery to inhibit host cellular protein translation (Raaben et al., 2007; Lindquist et al., 2010, 2011). SGs were not detected during infection by influenza A virus (IAV); however, a recombinant IAV lacking non-structural protein 1 (NS1), an inhibitor of PKR, efficiently induced the formation of SGs and the production of IFN-β in a PKR-dependent manner (Khaperskyy et al., 2012; Mok et al., 2012; Onomoto et al., 2012). In this case, SGs were suggested to play an important role as the sites of viral RNA sensing and subsequent anti-viral responses, because RLRs localized together with viral nucleoproteins and RNA as well as anti-viral proteins in SGs (Onomoto et al., 2012; Ng et al., 2013).

The RNA species produced during the course of RNA viral replication, such as mRNA, dsRNA, and 5′-triphosphate (5′-ppp) RNA including leader, trailer, genome, and antigenome RNAs as well as defective interfering (DI) genomes, have been shown to trigger the production of type I IFNs (Yoneyama et al., 2004; Hornung et al., 2006; Pichlmair et al., 2006; Strahle et al., 2007; Hausmann et al., 2008; Baum et al., 2010; Baum and Garcia-Sastre, 2011; Kato et al., 2011; Marq et al., 2011; Davis et al., 2012; Bowzard et al., 2013; Weber et al., 2013; Runge et al., 2014; Schmolke et al., 2014). We and other groups have recently reported that recombinant viruses of Sendai virus (SeV), a prototype of the family *Paramyxoviridae*, in which the C proteins are knocked-out or mutated, generate dsRNA in infected cells at levels similar to the production of IFN-β (Takeuchi et al., 2008; Irie et al., 2010). Previous studies also reported that, in the cases of SeV and IAV, copyback (cb)- and internal deletion (id)-type DI genomes, respectively, rather than full-length viral genomes, preferentially associated with RIG-I and strongly induced the production of IFN-β (Baum et al., 2010; Baum and Garcia-Sastre, 2011).

In spite of the large number of studies conducted in this field, it remains unknown what kinds of viral RNA species are recognized by RLRs and where the sites of recognition are in real infections by RNA viruses. In the present study, we compared some types of IFN-β-non-inducing and IFN-β-highly inducing viruses in the context of SeV infection. One of the biggest advantages of this study is that the comparison can be performed within the context of the same viral species. We found that some types of unusual RNA species that were distinguishable according to specific detectability by the anti-dsRNA antibody or FISH analysis were produced during the viral replication of IFN-inducing SeVs, but not IFN-non-inducing SeV strains, and accumulated into distinct cytoplasmic structures in an RNA-type-dependent manner. These unusual RNAs exhibited distinct properties in infected cells in terms of encapsidation with the viral N protein and subcellular distribution with SG marker proteins and RLRs. Our results suggest that RNA-type-dependent mechanisms recognize and accumulate virus-derived, IFN-β-inducible, unusual RNAs into specific compartment to trigger the production of IFN-β, and that SeV may evade detection by the host innate immune system by preventing the production of these RNA species.

## Materials and Methods

### Cells, Viruses, and Plasmids

LLC-MK2 cells (macaque monkey kidney-derived cells, described in Kiyotani et al., 1990) and HeLa cells (CCL-2; purchased from ATCC) were maintained as described previously (Irie et al., 2012). All of the SeVs, even the WT of strain Z and Hamamatsu (HMT), used in this study were recovered from cDNA using a reverse genetics technique as described previously (Kato et al., 1996; Fujii et al., 2002), except for the Cantell (CNT; VR-907; ATCC), Fushimi (FSM), and Nagoya (NGY) strains. The SeV recombinants, C′/C(-), 4C(-), and V(-), were kindly provided by Kato (National Institute of Infectious Diseases, Japan; Kato et al., 1997; Kurotani et al., 1998). All of the SeVs as well as virulent and avirulent Newcastle disease virus (NDV) Miyadera and D26 strains (Toyoda et al., 1987), respectively, were propagated in embryonated chicken eggs. SeV and NDV titers were determined by an immunofluorescent infectious focus assay in LLC-MK2 cells and expressed as cell infectious units (CIUs)/ml, as described previously (Kiyotani et al., 1990). One-step growth kinetics of the viruses was determined as described previously (Irie et al., 2014). Plasmids encoding the P, C, and V proteins of SeV strain Z in the pCAGGS.MCS vector have been described previously (Sakaguchi et al., 2005, 2011; Irie et al., 2008b, 2012).

### Antibodies

The polyclonal antibodies (pAbs) against whole virions of SeV and NDV were described previously (Kiyotani et al., 1990). The pAbs against the SeV P and C proteins were kindly provided by Kato. The monoclonal antibody (mAb) against the SeV N protein was kindly provided by Suzuki (National Institute of Infectious Disease, Japan). The mAb and pAb against G3BP1 (sc-365338, Santa Cruz Biotechnology; and ab39533, Abcam, respectively), pAb against RIG-I (28137; Immuno-Biological Laboratories, Japan), mAb against TIAR, and dsRNA (#8509, Cell Signaling Technology; and J2, Scicons, Hungary, respectively) were used according to the protocols of the suppliers.

### Sodium Arsenite and IFN-α Treatment

HeLa cells cultured on glass coverslips were infected with the indicated viruses or transfected with the indicated plasmids. The culture medium was replaced by serum-free DMEM 24 h post-infection (p.i.) or post-transfection (p.t.), and cells were treated with sodium arsenite (NaAsO_2_; Sigma) at a final concentration of 0.5 mM for 30 min or with IFN-α (1,000 IU/ml; R&D Systems) for 6 h.

### Immunofluorescence Microscopy

HeLa cells cultured on glass coverslips were transfected with the indicated plasmids using FuGENE HD transfection reagent (Promega) or infected with the indicated viruses at an MOI of 5. At 24 h p.t. or p.i., cells were fixed and immunostained as described previously using appropriate combinations of primary and secondary antibodies (Irie et al., 2013). To detect RIG-I, and dsRNA, the Tyramide signal amplification (TSA) kit with HRP-Goat Anti-Mouse IgG and Alexa Fluor 488 Tyramide (Molecular Probes) were used to increase the detection sensitivity. Coverslips were mounted on glass slides with the SlowFade Gold antifade reagent with or without DAPI (Molecular Probes) and observed using an LSM 5 confocal microscope (Carl Zeiss).

### RNA Preparation

HeLa cells were infected with the indicated viruses at an MOI of 5. At 24 h p.i., total RNA was prepared using the High Pure RNA Isolation Kit (Roche Diagnostics). Viral RNA in the working viral stocks was prepared using the High Pure Viral RNA Kit (Roche Diagnostics).

### Quantitative RT-PCR

Quantitative (q) RT-PCR was performed as described previously (Irie et al., 2010, 2014). qRT-PCR samples were analyzed using the Eco Real-Time PCR System (Illumina). For direct comparison, all of the indicated samples were analyzed in the same experiments. To detect the genome-length viral RNAs and cbDI genomes of SeV stocks, qRT-PCR was performed using the primer sets of 5SeVZ1683 + 3SeVZ1843, as described previously (Irie et al., 2008a, 2014), and 5cbDIdetect15,312-15,293 + 3cbDIdetect15,033-15,014, which were complementary to the regions of the indicated positions of SeV genome RNA, as described recently by Baum et al. (2010).

### Immunoprecipitation

HeLa cells were lysed in RIPA buffer (0.5% NP-40, 20 mM Tris-HCl [pH 7.4], 150 mM NaCl) after 24 h of infection by the indicated viruses or 30 min of the arsenite treatment, and the insoluble fraction was removed by high-speed centrifugation. The viral proteins in the lysates were removed by three consecutive immunoprecipitation steps using anti-SeV or anti-NDV pAbs and Protein G Sepharose beads (GE Healthcare Life Sciences). Supernatants were harvested from the final immunoprecipitation samples, and were then subjected to RNA preparation, as described above.

### RNA Transfection

HeLa cells cultured on glass coverslips were transfected with 1 μg of the indicated RNA samples prepared above, together with 0.5 μg of an empty pUC19 vector, using the FuGENE HD transfection reagent.

### Preparation of the CNT-lowDI Viral Stock

Cantell samples (1.4 × 10^9^ CIU/ml) that were 10^8~9^-fold serially diluted were inoculated into the allantoic cavity of 10-days-old embryonated chicken eggs, and incubated for 72 h at 34°C. Allantoic fluid was harvested from the eggs, and the titers of each fluid stock were determined as described above. RNA samples were prepared from 100 μl of each fluid stock, and the ratios of the cbDI genomes to viral genomes were determined by qRT-PCR as described above.

### Fluorescence *In Situ* Hybridization (FISH)

HeLa cells cultured on glass coverslips were infected with the indicated viruses. At 24 h p.i., cells were fixed with 3% paraformaldehyde solution in PBS, and then subjected to FISH analysis using the FISH Tag RNA Green Kit with Alexa Fluor 488 dye (Molecular Probes) according to the protocol of the supplier. The RNA probe was designed to be complementary to the region of 14,761–15,384 in SeV genome RNA. After FISH, some samples were further subjected to fluorescent immunodetection as described above using the indicated antibodies. Final samples were observed using an LSM 5 confocal microscope.

## Results

### Strong Correlations between Levels of the Induction of IFN-β and the Formation of G3BP1-Positive Granules by Infection of C-Mutated and -Deficient SeV Recombinants

**FIGURE 1:**
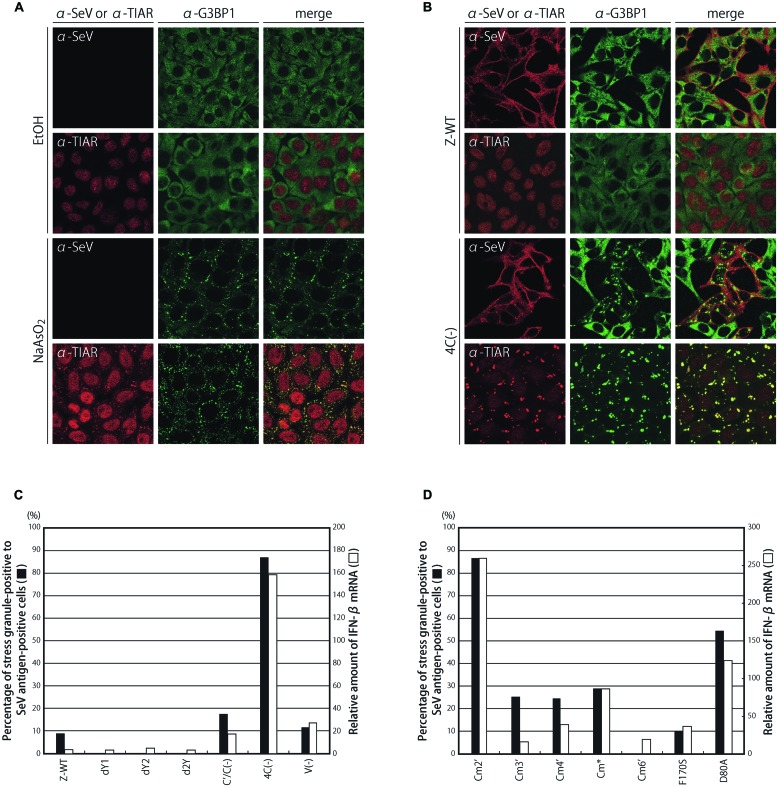
**Subcellular distribution of G3BP1 and TIAR in HeLa cells treated with or without sodium arsenite **(A)**, and infected with a series of C-deficient rSeVs, dY1, dY2, d2Y, C′/C(-), and 4C(-), and a V-deficient rSeV, V(-) (B)**. HeLa cells treated with ethanol (EtOH) or sodium arsenite (NaAsO_2_) or infected with the indicated virus were immunostained with anti-G3BP1 mAb, anti-TIAR mAb, and anti-SeV pAb. **(C)** The G3BP1 foci in the samples of **(B)** were observed under a fluorescent microscope, and the number of G3BP1 granule-positive cells was counted and is presented as percentages against the number of SeV antigen-positive cells (closed bars). The relative amounts of IFN-β mRNA in cells infected with the indicated viruses were determined by qRT-PCR, and normalized to those of β-actin mRNA (open bars). **(D)** Similar experiments were performed for a series of C-mutated SeV recombinants, and the results are presented as bar graphs, similarly to **(C)**.

We first examined whether G3BP1-positive granular structures were formed during infections by a series of C knocked-out and mutated SeV recombinants and the parental Z strain by immunofluorescence microscopy (**Figure 1**; Supplementary Figure S1).

Treating HeLa cells with sodium arsenite (NaAsO_2_) caused the formation of granular structures in the cytosol, which were defined as SGs based on the expression of related proteins such as G3BP1 and TIAR. G3BP1 and TIAR are well-established SG-associated proteins that are typically and diffusely present throughout the cytoplasm and dominantly present in the nucleus, respectively. However, treating cells with arsenite markedly changed the localization to form SGs containing these proteins in nearly all cells (**Figure 1A**).

When cells were infected with 4C(-), G3BP1-positive granular structures were observed in almost 90% of SeV antigen-positive cells, and were considered to be SG-like structures since TIAR was also detected in the majority of the granules (**Figures 1B,C**). In this situation, TIAR was mostly in the cytoplasmic structures, whereas a larger part of TIAR was still observed in the nucleus in the arsenite-treated cells. This difference is probably due to the different exposure time to the stimuli: 30 min. for the treatment with arsenite and 24 h for the infection. In contrast, the percentages were only 8% or less in cells infected with the parental Z-WT as well as the dY1, dY2, and d2Y recombinants, which lacked the smaller C proteins, Y1, Y2, and both Y1 and Y2, respectively (**Figure 1C**; Supplementary Figure S1A). An infection by C′/C(-) and V(-), lacking the larger C proteins, C′ and C, and the V protein, respectively, resulted in a slight increase in the number of granules (**Figure 1C**; Supplementary Figure S1A). Of note, unlike the viruses reported previously, such as NS1-deficient IAV and vesicular stomatitis virus (Mok et al., 2012; Onomoto et al., 2012; Dinh et al., 2013), the fluorescence of the SeV antigen was not colocalized with that of the representative SG marker G3BP1 in the granules (**Figure 1B**; Supplementary Figure S1).

IFN-β mRNA levels in the infected cells were also compared between the viruses (**Figure 1C**). Strong correlations were observed between IFN-β mRNA levels and the percentages of granular structure-forming cells against infected cells. Similar strong correlations were observed for a series of C mutant viruses that possessed single- to triple-amino-acid substitutions of highly conserved, charged amino acids within the C proteins, which diminished their ability to antagonize the host IFN system to various degrees (**Figure 1D**; Supplementary Figure S1B; Irie et al., 2010).

The marked difference observed in granular formation and IFN-β mRNA levels among the viruses was not due to their different growing abilities. The one-step growth kinetics of all the recombinants used above was previously reported to be similar to that of the parental Z-WT, except for 4C(-) and C′/C(-), the titers of which were 1–2 logs lower than that of Z-WT throughout the time course, and viral protein synthesis in the infected cells did not significantly differ among the viruses examined (Kurotani et al., 1998; Irie et al., 2008a, 2010). Treatment of HeLa cells with IFN-α did not induce G3BP1-positive granules, indicating that the formation of SG-like structures observed by SeV infection was triggered by SeV infection, but not by SeV-induced type I IFNs (Supplementary Figure S1C).

Taken together, these results strongly suggested that a relationship may exist between the formation of G3BP1-positive granules and the induction of IFN-β in the C recombinants, and also that C proteins may suppress the formation of granules.

### SeV could not Inhibit the Arsenite- and Virus-Triggered Formation of G3BP1-Positive Granules

**FIGURE 2:**
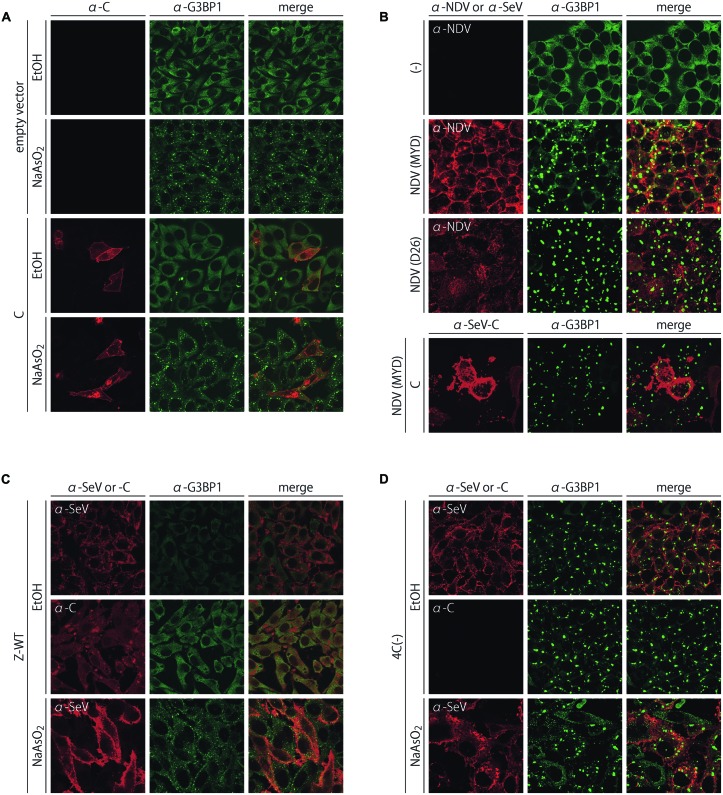
**Subcellular distribution of G3BP1 after the treatment with arsenite **(A)** and the infections with the virulent and avirulent NDV strains, MYD and D26, respectively **(B)**, in HeLa cells that received an empty pCAGGS.MCS vector or pCAGGS-C. (C,D)** Subcellular distribution of G3BP1 after the treatment with arsenite in HeLa cells infected with SeV-Z-WT **(C)** or 4C(-) **(D)**. Cells were immunostained with anti-G3BP1 mAb together with anti-SeV, anti-NDV, or anti-SeV C pAbs.

We examined whether expression of the C protein alone and infection of the non-granule-forming SeV could inhibit the formation of granules in cells treated with arsenite or infected with NDV (**Figure 2**; Supplementary Figure S2). NDV, another prototypic paramyxovirus, which was considered to be an IFN-β-inducing virus, could induce G3BP1-positive granules in nearly all cells infected with virulent as well as avirulent strains (Miyadera and D26 strains, respectively; **Figure 2B**).

The C protein alone failed to inhibit the formation of granules in cells treated with arsenite (**Figure 2A**) as well as infected with NDV (**Figure 2B**), although the number of granules was slightly reduced in the C-expressing cells treated with arsenite compared to that observed in the neighboring non-C-expressing cells (**Figure 2A**). In addition to C, the other P gene products, P and V proteins, also failed to inhibit the formation of both types of granule (Supplementary Figure S2). Infection by SeV-Z-WT and C-deficient recombinant 4C(-) also failed to inhibit the arsenite-induced formation of granules (**Figures 2C,D**). Small granules dispersed in the cytoplasm were induced by arsenite in both cases of infection by WT and 4C(-). Of note, the G3BP1-positive granules induced by arsenite and viruses, such as 4C(-) and NDV, differed in size; the granules induced by the infection were apparently larger than those induced by arsenite (**Figures 1** and **2**), implying a possible difference in cellular pathways leading to these two types of granular structure. These results demonstrated that SeV did not have the ability to inhibit the formation of both types of granules.

### The Induction of IFN-β in Infections by SeV Cantell Strain was not Related to the Formation of SG-Like Structures

**FIGURE 3:**
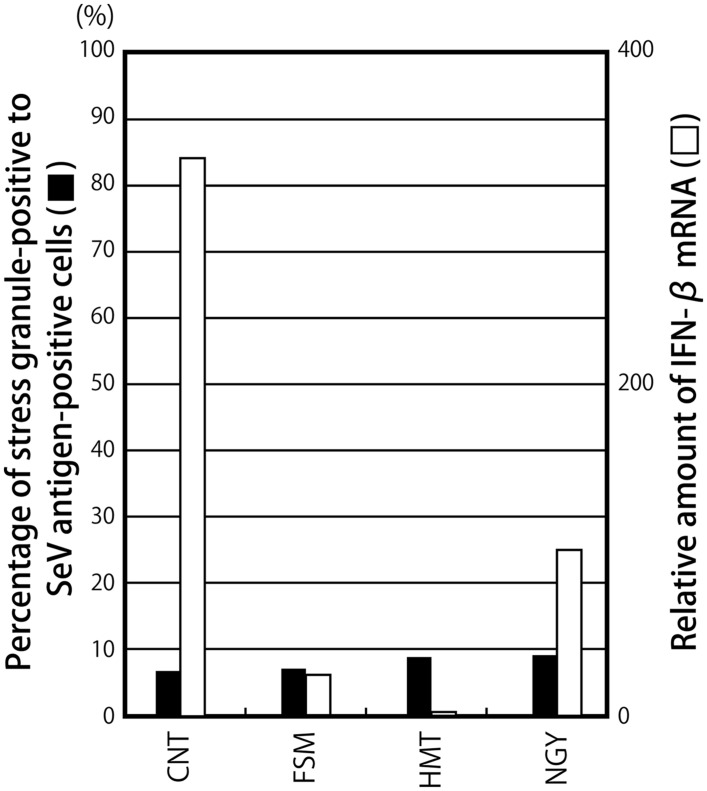
**The percentages of G3BP-positive granules against SeV antigen-positive cells and the relative amounts of IFN-β mRNA are presented as bar graphs, similarly to **Figure 1C****.

A marked difference was noted in the abilities of the SeV strains to induce IFN-β. Although most of the SeV strains including Z have been characterized by their strong ability to counteract the innate immune system, the CNT strain has been widely used as a virus that induces high levels of IFN-β (Baum et al., 2010; Tapia et al., 2013). Therefore, we compared the abilities of some SeV strains to induce IFN-β and SG-like structures (**Figure 3**; Supplementary Figure S3).

In all cases, G3BP1-positive granular structures were only detected in less than 9% of SeV antigen-positive cells (**Figure 3**; Supplementary Figure S3A). IFN-β mRNA was not highly induced in cells infected with the SeV strains, except for CNT, whereas CNT induced IFN-β mRNA at a level that was 46-fold higher than that by Z (**Figure 3**). Regarding the SeV strains, unlike that observed in the C recombinants, a correlation was not observed between IFN-β mRNA levels and the percentage of granular structure-forming cells against infected cells (**Figure 3**). The different levels of IFN-β mRNA induced by the strains could not be attributed to differences in viral growth (Supplementary Figure S3B).

These results suggested that the formation of G3BP1-positive granules was not necessarily required to sense the SeV CNT infection, followed by the production of IFN-β, unlike the C recombinants.

### Non-Encapsidated Viral RNA Species, but not the Encapsidated cbDI Genome, was a Potent Inducer of G3BP1-Positive Granules

**FIGURE 4:**
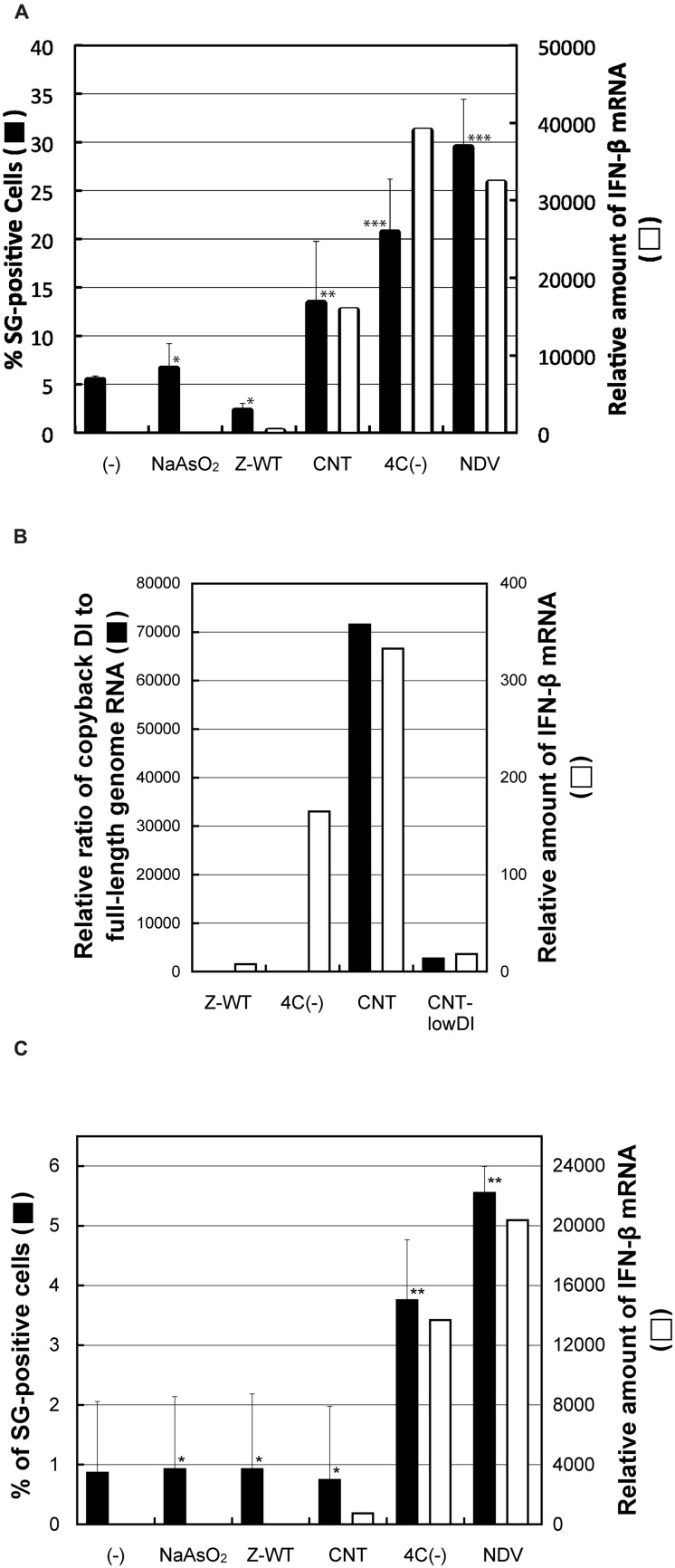
**(A)** The percentages of G3BP1 granule-positive cells against the total number of cells that received total RNA samples prepared from HeLa cells treated with or without arsenite or infected with SeV-Z-WT, SeV-CNT, SeV-4C(-), or NDV-MYD in an average of 20 random microscopic fields are presented as bar graphs (closed bars). The calculated *P* values were: arsenite, *P* = 0.49; Z-WT, *P* = 0.15; CNT, *P* = 0.031; 4C(-), *P* = 0.0023; NDV, *P* = 0.0055. The relative amounts of IFN-β mRNA in HeLa cells that received the RNA samples were determined by qRT-PCR, and normalized by those of β-actin mRNA. **(B)** The amounts of cbDI genomes as well as genome-length viral RNAs in HeLa cells infected with the SeVs, Z-WT, 4C(-), CNT, or CNT-lowDI, were determined by qRT-PCR. The relative ratio of the cbDI genomes to the genome-length viral RNAs are presented as bar graphs. The relative amounts of IFN-β mRNA in HeLa cells that infected with the indicated viruses were determined, as performed in **(A)**. **(C)** RNA samples were prepared from the viral protein-removed cell lysate and introduced into HeLa cells. Percentages of G3BP1 granule-positive cells against the total number of cells in an average of twenty random microscopic fields are presented as bar graphs, as performed in **(A)**. The calculated *P* values were: arsenite, *P* = 0.97; Z-WT, *P* = 0.96; CNT, *P* = 0.42; 4C(-), *P* = 0.033; NDV, *P* = 0.027. *P > 0.1; **P < 0.05; ***P < 0.01. The relative amounts of IFN-β mRNA in HeLa cells that received the RNA samples were determined, as performed in **(A)**.

We attempted to identify the reason for the difference in granular formation between these two types of IFN-β-inducing virus, 4C(-) and CNT. To address this issue, we first examined the ability of total RNA prepared from virus-infected as well as arsenite-treated cells to induce G3BP1-positive granules (**Figure 4A**; Supplementary Figure S4A). Total RNA samples prepared from cells infected with any of the IFN-β-inducing viruses, SeV-CNT, 4C(-), and NDV, were able to induce the granules as well as IFN-β in HeLa cells despite no formation of the granules by the CNT infection, whereas those from cells infected with the IFN-β-non-inducing SeV-Z-WT or treated with arsenite were not (**Figure 4A**).

We then examined the content rates of cbDI genomes, a potent ligand for RIG-I, against viral genome-length RNAs in the RNA samples used above (**Figure 4B**). The CNT sample contained cbDI genomes at a level that was ~15,700-fold higher than that in the Z-WT sample, although the sample of 4C(-) contained similar levels to the Z-WT sample (**Figure 4B**). We further examined the effect of removal of encapsidated viral RNA species, such as viral full-length and DI genomes, by three consecutive rounds of immunoprecipitation using antisera against the whole virions of SeV and NDV prior to the preparation of RNA samples (Supplementary Figure S4B). Total RNA prepared from the post-immunoprecipitation samples of 4C(-) and NDV was still able to induce the granules as well as IFN-β, but that of CNT lost these abilities (**Figure 4C**). Since the naked cbDI genomes have been reported readily to form an ideal structure as the RIG-I ligands of 5′-triphosphated, blunt-ended dsRNA (Kolakofsky, 1976), these results indicated that the major IFN-β-inducing viral RNA species produced in the cells infected with CNT was encapsidated cbDI genomes, whereas those for SeV-4C(-) and NDV were not.

### SeV-Induced G3BP1-Positive Granules Included RIG-I, but not J2-Detectable Viral dsRNA

**FIGURE 5:**
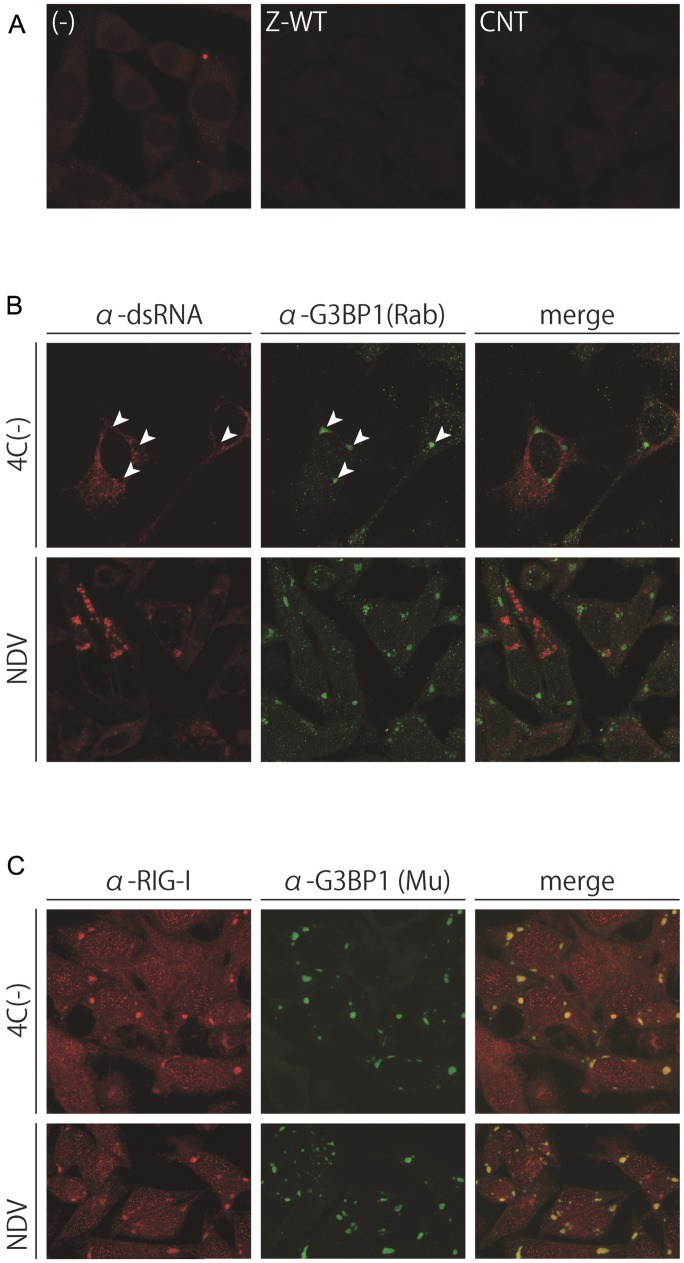
**(A)** dsRNA was not detected by the anti-dsRNA antibody, J2. HeLa cells uninfected, (-), or infected with Z-WT or CNT were immunostained with the J2 antibody. **(B,C)** Subcellular distribution of J2-detectable dsRNA and G3BP1 **(B)** or RIG-I and G3BP1 **(C)** in HeLa cells infected with 4C(-) or NDV-MYD. Cells were immunostained with the J2 antibody and anti-G3BP1 pAb **(B)** or anti-RIG-I pAb and anti-G3BP1 mAb **(C)**. Arrowheads in **(B)** indicate the sites of G3BP1-positive granules. The signals of the J2 and RIG-I antibodies were amplified using a TSA kit.

**FIGURE 6:**
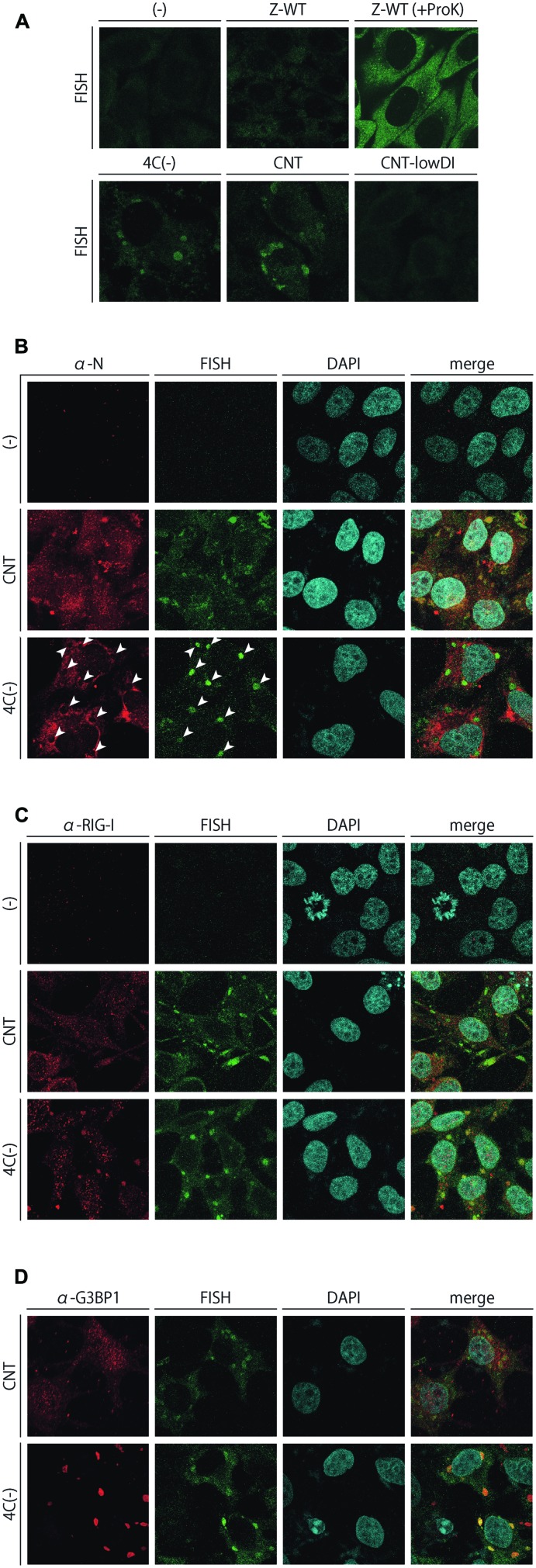
**(A)** FISH analysis of HeLa cells infected with the SeVs, Z-WT, 4C(-), CNT, and CNT-lowDI. HeLa cells were infected with the indicated viruses. After the fixation and permeabilization of cells, they underwent hybridization with an Alexa Fluor 488-labeled RNA probe complementary to the region of 14,761–15,384 nt in the (-)-sense SeV genome, with or without being treated with Proteinase K at a final concentration of 50 μg/ml for 5 min. **(B–D)** Subcellular colocalization of FISH signals with SeV N **(B)**, RIG-I **(C)**, and G3BP1 **(D)**. The FISH samples prepared in **(A)** were immunostained further with an anti-SeV N mAb, anti-RIG-I pAb, or anti-G3BP1 mAb. Signals for RIG-I were amplified using the TSA kit. The arrowheads in **(B)** indicate the sites of FISH-positive inclusions.

The results above suggested that there may be at least two types of IFN-β-inducing viral RNA species with a marked difference in the formation of SG-like granules. To elucidate this difference, we examined the subcellular distribution of unusual viral RNA species produced by 4C(-) and CNT (**Figures 5** and **6**).

We and other groups previously reported a strong correlation between the production of dsRNA detected by the anti-dsRNA antibody J2 (J2-dsRNA) and the induction of IFN-β in infections of the C-mutated SeV recombinants and NDV (Takeuchi et al., 2008; Irie et al., 2010). Therefore, we first examined the production and subcellular distribution of J2-dsRNA in the cells infected with the IFN-β-inducing viruses by immunofluorescence microscopy (**Figure 5**). DsRNA fluorescent signals were absent in cells infected with IFN-non-inducing Z-WT as well as in those with the IFN-β-inducing strain CNT (**Figure 5A**). In contrast, dsRNA fluorescent signals were clearly observed in the cytoplasm of cells infected with IFN-β-inducing SeV-4C(-) and NDV with dispersed and granular distributions, respectively (**Figure 5B**).

A previous study reported that NS1-deficient IAV infections caused RIG-I to form granular aggregates that contained SG markers as well as viral RNA (Onomoto et al., 2012). Therefore, the cells infected with 4C(-) and NDV were co-stained with anti-RIG-I and anti-G3BP1 antibodies (**Figure 5C**). Similar to IAV, RIG-I almost perfectly colocalized with the virus-induced G3BP1-positive granules in both cases of infection (**Figure 5C**); however, unlike IAV, these granules did not colocalize with the viral antigen or viral J2-dsRNA (**Figure 5B**, arrowheads), as shown in **Figures 1** and **2**. Together with the results of **Figure 4**, J2-dsRNA appeared to be not or less encapsidated by viral nucleoproteins because the access of the antibody to and the formation of dsRNA by tightly encapsidated viral RNA, such as viral genomes, was unlikely.

### Non- and Partially Encapsidated Unusual Viral RNA Species were Selectively Incorporated into avSG-Like and Non-avSG-Like Inclusions

We further attempted to visualize the subcellular distribution of the unusual viral RNA species that were not fully encapsidated, unlike the genome-length viral RNAs, by FISH analysis (**Figure 6**). Infected cell samples were prepared without a protease treatment to exclude fully encapsidated viral RNA species, and were then stained with an RNA probe complementary to the 14,761–15,384 region of the (-)-sense viral genome RNA, designed by referring to two well-characterized cbDI genomes of SeV (Calain et al., 1992; Martinez-Gil et al., 2013). Fluorescence-positive cytoplasmic inclusions were observed in the 4C(-)- as well as in the CNT-infected samples, while an apparent signal was not detected in Z-WT-infected or uninfected samples (**Figure 6A**). When the Z-WT-infected sample was treated with Proteinase K, apparent signals were observed throughout the cytoplasm (**Figure 6A**). These results strongly suggested that fully encapsidated viral genomes were not detected, whereas non- or partially encapsidated viral RNA species were detectable in this system. The FISH-positive inclusions observed in CNT-infected cells were no longer detected in the cells infected with the CNT-lowDI (**Figure 6A**), which contained ~10-fold fewer cbDI genomes than the original CNT sample (**Figure 4B**). Together with the results of **Figure 4**, the FISH signals observed in the CNT-infected cells were considered as the cbDI genomes.

The FISH samples of 4C(-) and CNT-infected cells were further immunostained to examine the subcellular colocalization of FISH signals, the SeV N protein, RIG-I, and G3BP1 (**Figures 6B–D**, respectively). The SeV N protein was detected in the FISH-positive inclusions of CNT-infected cells, suggesting that the RNA species detected in the CNT samples were only partially, not fully, encapsidated, unlike the fully encapsidated, full-length, intact viral genomes (**Figure 6B**). In contrast, the N protein was not detected in the inclusions of 4C(-)-infected cells, which suggested that the RNA species detected in the 4C(-) samples were not encapsidated (**Figure 6B**). The FISH-positive inclusions of CNT-infected cells were not apparently colocalized with RIG-I or G3BP1, whereas most of those in the 4C(-)-infected cells colocalized obviously with RIG-I and G3BP1, unlike the J2-dsRNA (**Figures 6C,D**).

These results indicated that unusual viral RNA species harboring the 5′-region of (-)-sense SeV genome RNA, which was not produced during infection by IFN-β-non-inducing Z-WT, was produced in infections by IFN-β-inducing CNT and 4C(-), and were selectively formed into distinct cytoplasmic inclusions in an RNA-type-dependent manner. The inclusions in the 4C(-)-infected cells could be identified as avSGs, and may be the site to detect viral RNA by RIG-I, whereas those in CNT-infected cells were not avSGs, but as-yet-unidentified structures.

## Discussion

Although a number of studies have examined host innate immune responses against pathogenic microbes including RNA viruses, the virus-derived RNA species that serve as PAMPs in real viral infections and the sites at which PAMPs are recognized by RLRs have yet to be clarified in detail. Two important insights were reported recently. Regarding the real PAMPs in infections by RNA viruses, the cb and id types of DI genomes were identified as the ligands of RIG-I in SeV- and IAV-infected cells, respectively, both of which could form ideal structures as the RIG-I ligands (Baum et al., 2010; Baum and Garcia-Sastre, 2011; Martinez-Gil et al., 2013). The SG-like structures have been suggested to serve as the sites at which the RLRs encounter viral RNA and subsequently activate the IFN signaling pathways in infections by RNA viruses (Onomoto et al., 2012; Yoo et al., 2014). In order to establish what and where viral RNA species were detected by RLRs, in the present study, we compared two types of IFN-β-inducing SeV, a recombinant 4C(-) and a strain CNT, with a non-IFN-β-inducing Z strain, in terms of the formation of SG-like granules and the production of unusual viral RNA species. A major advantage of our study is that the comparison can be performed within the context of the same viral species.

Several types of unusual viral RNA species were found to be generated in cells infected with the IFN-inducing SeVs but not those with the IFN-non-inducing SeVs (summarized in **Table 1**). One was a dsRNA (J2-dsRNA) that was detected by the anti-dsRNA antibody, J2 (type I in **Table 1**). As for SeV, the generation of J2-dsRNA may have been restricted by the C proteins because it was only detected in cells infected with C-deficient or mutated recombinants, but not in those with intact SeVs (**Figure 5**; Takeuchi et al., 2008; Irie et al., 2010). We and other groups demonstrated that J2-dsRNA activated PKR, and this was followed by the phosphorylation of eIF2 and the production of IFN-β, both of which resulted in antiviral effects in the host cells. The activation of PKR has been reported to induce the formation of SG-like structures during infections by some RNA viruses, such as IAV and measles virus (MeV; Mok et al., 2012; Onomoto et al., 2012; Okonski and Samuel, 2013), and this also appears to be the case for the SeV C recombinants. Unlike these viruses, the SG-like structures formed during 4C(-) infections did not include the J2-dsRNA, which was dispersed in the cytoplasm, although they contained RIG-I (**Figure 5**). This may lead to the assertion that the SG-like structures induced during infections by C recombinants are not the sites at which to detect SeV infections.

**Table 1:** Properties of unusual viral RNA species produced by infections of the IFN-β-inducing SeVs.


Type	SeV	Defined as	Detected by			Colocalized with			Encapsidated with N	Accumulated into cytoplasmic structures	Viral protein responsible for the production
			J2	FISH		SG markers	SeV N	RIG-I			
I	4C(-)	dsRNA	+	-		-	-	-	No	No, dispersed in the cytoplasm	C
II	4C(-)	Unknown	-	+		+	-	+	No	Yes, avSG-like structures	C
III	CNT	cbDI	-	+		-	+	-	Yes, but not fully	Yes, undefined, non-avSG structures	ND

*ND, not determined.*

However, the SG-like structures formed by 4C(-) were revealed to contain another type of unusual viral RNA species by FISH analysis, in which an RNA probe targeting the 600 nt region of the 5′-end of the (-)-sense SeV genome was used (type II in **Table 1**; **Figure 6**). This now strongly suggests that the SG-like structures found in the 4C(-) infection are defined as avSGs. Unlike the IAV infection, the type I and II RNA species seemed to be not or less encapsidated, given that they were not colocalized with viral antigens and were not removed from the infected cell lysates by immunoprecipitation using anti-SeV antibody (**Figures 1** and **4–6**). SeV trailer RNA, which is transcribed from the 3′-ends of (+)-sense antigenome RNA, was previously reported to interact with TIAR to inhibit apoptosis and the formation of SGs induced by infection (Iseni et al., 2002). The 4C(-) virus was shown to induce apoptosis more quickly and severely in infected cells than the WT virus (Irie et al., 2010). Although appearing to contain the non-encapsidated 5′-end of a (-)-sense genome RNA, at least in the part that is concordant with the trailer RNA, it is unlikely that the type II RNA observed in the 4C(-)-infected cells has the ability of the trailer RNA to inhibit apoptosis and SG formation. Although details of the type I and II RNAs remain to be solved, given the cytoplasmic replication of SeV RNA without forming inclusion bodies and the differences of the RNA species in subcellular distribution and reactivity with the J2 and FISH probe, the type I dsRNA somewhat unwound actively or incidentally into the type II RNA might be accumulated into the avSGs.

Although the SG-like structures and J2-dsRNA were not detected during CNT infections, FISH-positive non-granular-shaped inclusions were observed (type III in **Table 1**; **Figure 6**). These CNT inclusions were markedly different from those of 4C(-). The inclusions did not include RIG-I or G3BP1, but contained the N protein (**Figure 6**), suggesting that the inclusions were not avSGs, and that the type III RNA was at least partially encapsidated. The CNT stock used in the present study contained a larger amount of cbDI genomes than the other viral stocks (**Figure 4B**). The SeV cbDI genomes have been shown to be partially and/or more loosely encapsidated than intact genomes (Kolakofsky, 1976; Strahle et al., 2006). The SeV cbDI genomes were recently identified as strong ligands for RIG-I (Baum et al., 2010; Tapia et al., 2013). Indeed, the CNT-lowDI had lost the ability to induce IFN-β and the inclusions had not been found in the infected cells (**Figures 4B** and **6**). Taken together, the type III RNA was identified as the cbDI genome. These results indicated that the process of detecting infections and the subsequent induction of IFN-β differed largely between 4C(-) and CNT: avSG-dependent and -independent mechanisms for 4C(-) and CNT, respectively.

Most viruses have been shown to possess the ability to antagonize host IFN pathways in order to avoid activating host antiviral actions, and this strategy is mostly based on a counteraction against the molecules involved in these pathways (Versteeg and Garcia-Sastre, 2010). However, a recent study reported that encephalomyocarditis virus (EMCV) has the ability to disrupt SGs by cleaving G3BP1 in order to avoid the innate immune detection of its infection and subsequent induction of IFN-β (Ng et al., 2013), suggesting that another effective strategy for viral evasion from the IFN system by preventing avSG formation exists. Generation of the unusual viral RNA species triggering the production of IFN-β seems to be suppressed during intact RNA viral replication. Similarly to SeV, another paramyxovirus MeV C protein was recently shown to impair the production of J2-dsRNA and the activation of PKR coupled with the formation of SG-like structures (Okonski and Samuel, 2013; Pfaller et al., 2014). Unlike the case of SeV, in which the knockout of C resulted in the production of J2-dsRNA (**Figures 5** and **6**), but not cbDI genomes, the J2-dsRNA produced during the infection by a C-deficient MeV recombinant was reported to be a cbDI genome (Pfaller et al., 2014). Although the cbDI genomes were more dominantly produced by SeV-CNT than by the other strains and C-recombinants tested (**Figure 4B** and data not shown), this unique property of CNT might be attributed to its C protein that possibly have a functional difference with those of the other SeVs. The C proteins of both SeV and MeV have been shown to play critical roles in modulating viral RNA synthesis and maintaining its integrity by potentially stabilizing the ribonucleoprotein (RNP)-polymerase complex (Tapparel et al., 1997; Reutter et al., 2001; Bankamp et al., 2005; Irie et al., 2008a, 2014; Ito et al., 2013). Dysfunctions in the C proteins may result in a disturbance in integrity, leading to the production of the unusual, IFN-β-inducing RNA species.

## Conclusion

The results of the present study indicate that several types of IFN-β-inducible, unusual viral RNA species may be produced during SeV infections and included in avSG-like and non-avSG-like cytoplasmic inclusions, which suggests that RNA-type-dependent mechanisms recognize and accumulate such unusual viral RNAs in specific compartments. In addition, the production of these unusual RNA species may be restricted during intact viral replication in order to avoid detection by host innate immunity.

## Conflict of Interest Statement

The authors declare that the research was conducted in the absence of any commercial or financial relationships that could be construed as a potential conflict of interest.
