# Supplementary material for: IFN-β-inducing, unusual viral RNA species produced by paramyxovirus infection accumulated into distinct cytoplasmic structures in an RNA-type-dependent manner
Source: Front Microbiol. 2015 Aug 4;6:804. doi: 10.3389/fmicb.2015.00804 (PMC4523817; doi:10.3389/fmicb.2015.00804)
Supplement: Supplementary file 1 [file Presentation_1.PDF]

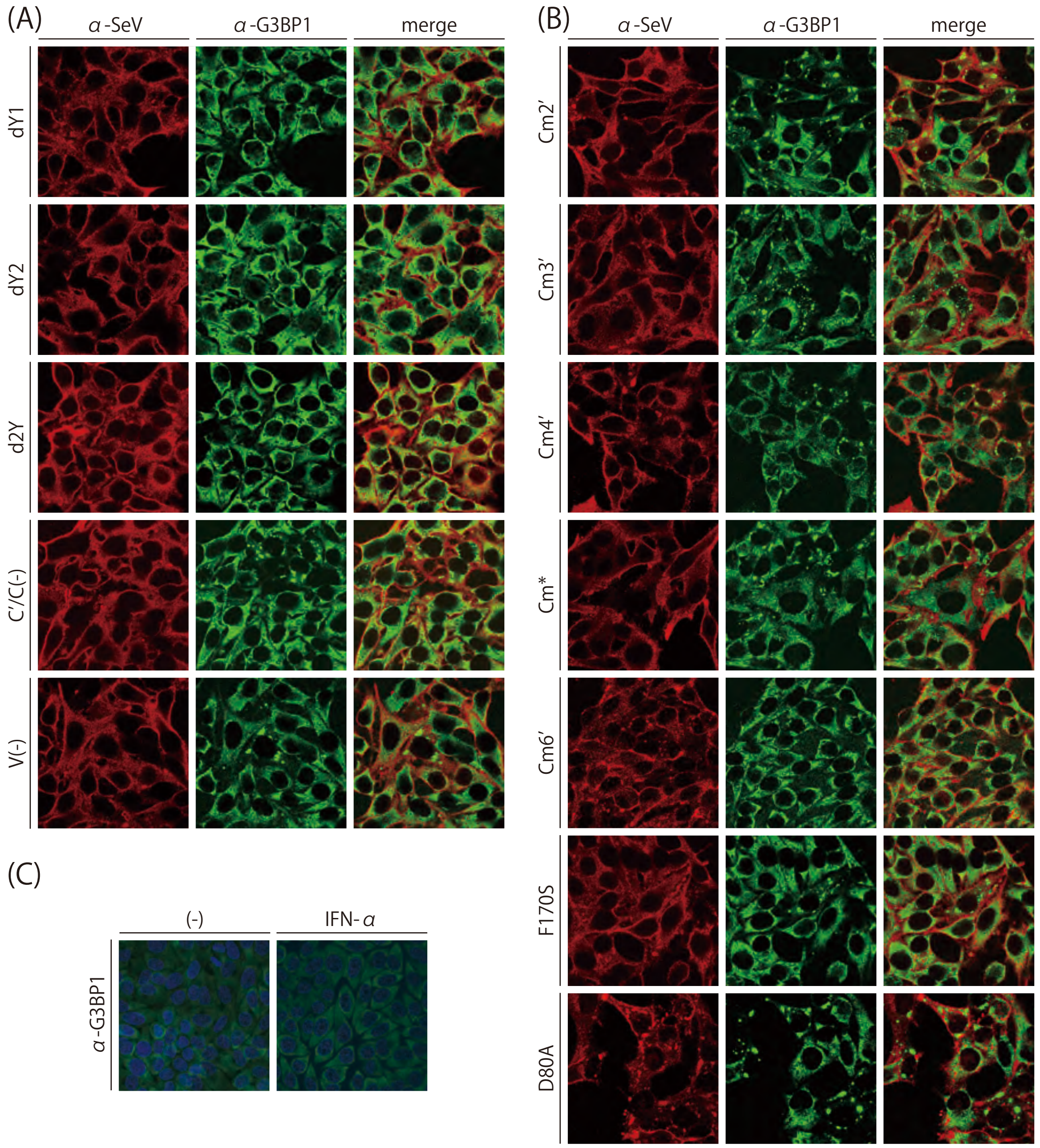

Figure S1. Subcellular distribution of G3BP1 in HeLa cells infected with a series of C-deficient rSeVs, dY1, dY2, d2Y, and 4C(-), and a V-deficient rSeV, V(-) (A), a series of C-mutated rSeVs, Cm2' , Cm3' , Cm4' , Cm*, Cm6' , F170S, and D80A (B), and treated with or without IFN-α (C). At 24 h p.i. or 6 h post-treatment, cells were immunostained with anti-G3BP1 mAb and anti-SeV pAb.

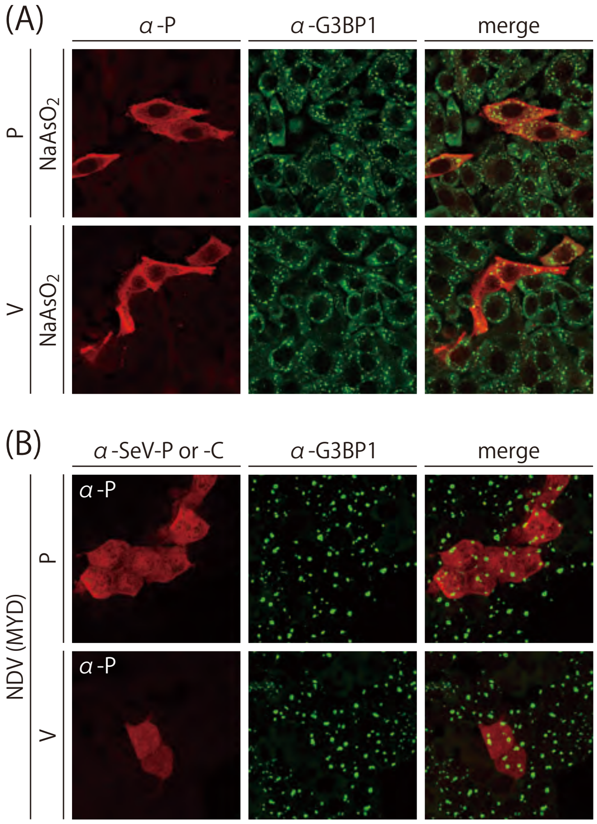

Figure S2. Subcellular distribution of B3BP1 after the treatment with arsenite (A) or the infection with NDV-MYD (B) in HeLa cells that received pCAGGS-P or -V. At 30 min post-treatment or 24 h p.i., cells were immunostained with an anti-G3BP1 mAb together with anti-SeV P or C pAb.

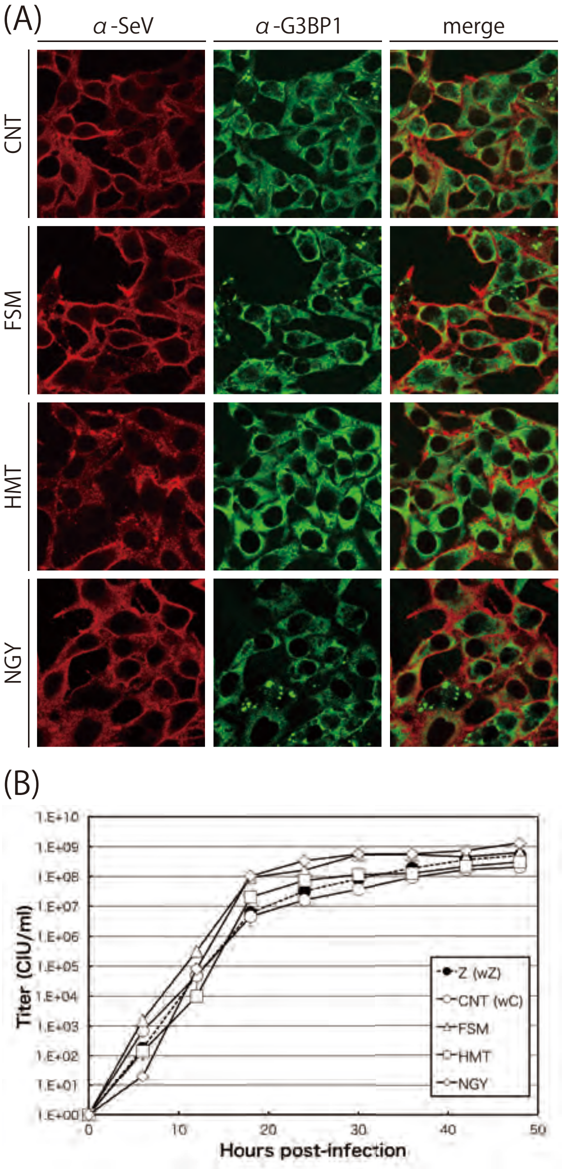

Figure S3. (A) Subcellular distribution of G3BP1 in HeLa cells infected with the SeV strain CNT, FSM, HMT, or NGY. At 24 h p.t., cells were immunostained with anti-G3BP1 mAb and anti-SeV pAb. (B) One-step growth curves of the indicated SeV strains on LLC-MK2 cells.

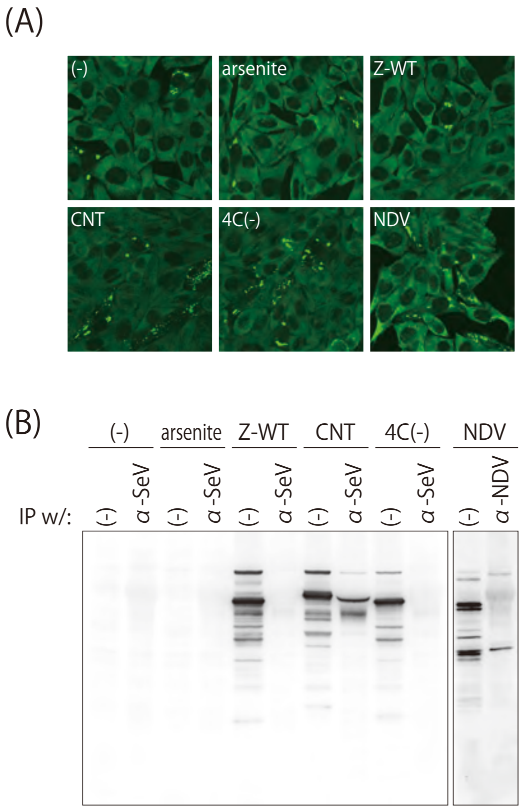

Figure S4. (A) Subcellular distribution of G3BP1 in HeLa cells that received total RNA samples prepared from HeLa cells treated with or without arsenite or infected with SeV-Z-WT, SeV-CNT, SeV-4C(-), or NDV-MYD. Cells were immunostained with an anti-G3BP1 mAb. (B) Detection of viral proteins in HeLa cell lysates after the removal of viral proteins by immunoprecipitation. HeLa cell lysates were prepared after the treatment with or without arsenite or infection with the indicated viruses. Cell lysates were subjected to three consecutive rounds of immunoprecipitation with anti-SeV or anti-NDV pAb to remove viral protein in the lysates or without the antibodies. The final lysates were analyzed by Western blotting using anti-SeV or anti-NDV pAb.
